# Nodal Metastases in Acinic Cell Carcinoma of the Parotid Gland

**DOI:** 10.3390/jcm8091315

**Published:** 2019-08-27

**Authors:** Stefan Grasl, Stefan Janik, Matthaeus C. Grasl, Johannes Pammer, Michael Formanek, Ilan Weinreb, Bayardo Perez-Ordonez, Andrew Hope, Ali Hosni, John R. de Almeida, Jon Irish, Ralph Gilbert, David P. Goldstein, Boban M. Erovic

**Affiliations:** 1 Department of Otorhinolaryngology and Head and Neck Surgery, Medical University of Vienna, 1090 Vienna, Austria; 2 Clinical Institute of Pathology, Medical University of Vienna, A-1097 Vienna, Austria; 3 Department of Otorhinolaryngology and Phonetics, Hospital of St. John of God, 1020 Vienna, Austria; 4 Sigmund Freud University, Medical School, 1020 Vienna, Austria; 5 Department of Pathology, University Health Network, Princess Margaret Cancer Centre, Toronto, ON M5G 2C4, Canada; 6 Department of Radiation Oncology, Princess Margaret Cancer Centre, University of Toronto, Toronto, ON M5G 2C4, Canada; 7 Department of Otolaryngology-Head and Neck Surgery, Princess Margaret Cancer Centre/University Health Network, University of Toronto, Toronto, ON M5G2C4, Canada; 8 Institute of Head and Neck Diseases, Evangelical Hospital Vienna, 1180 Vienna, Austria

**Keywords:** acinic cell tumor, parotid gland, elective neck dissection

## Abstract

The objective of this study was to evaluate the clinical outcome of patients with acinic cell carcinomas of the parotid gland after elective neck dissection (END). A retrospective chart review was performed including 66 patients with acinic cell carcinoma of the parotid gland. Clinical parameters were retrieved and statistically analyzed regarding disease-free survival (DFS) and disease-specific survival (DSS). An END was done in 27 (40.9%) patients, and occult metastases were detected in 4 (14.8%) patients of whom three were low-grade carcinoma. Positive neck nodes were associated with significantly worse DSS (*p* = 0.05). Intermediate and high-grade carcinoma (HR 8.62; 95% confidence interval (CI): 1.69–44.01; *p* = 0.010), perineural invasion (HR 19.6; 95%CI: 0.01–0.37; *p* = 0.003) and lymphovascular invasion (HR 10.2; 95%CI: 0.02–0.59; *p* = 0.011) were worse prognostic factors for DFS. An END should be considered in patients with acinic cell carcinoma of the parotid gland due to (i) a notable rate of occult neck metastases in low-grade tumors and (ii) the worse DSS of patients with positive neck nodes.

## 1. Introduction

Salivary gland malignancies (SGM) represent a group of heterogeneous cancers with distinct biological behavior and complex clinicopathological characteristics [1,2,3,4,5,6,7]. Due to the rarity of parotid gland malignancies as a whole, numerous clinical studies analyzed all forms of salivary gland cancers together without considering differences in the biology of individual histologic variants of SGMs. One of the major differences between the diverse malignancies is the risk of lymph node metastases. In addition to histology, the risk of lymph node metastases depends on the tumor location, grade, and tumor size [8,9,10,11,12,13,14]. Acinic cell carcinoma, one of the most common salivary malignancies, has a reported incidence of lymph node metastases of 10% with occult metastatic lymph node disease occurring in up to 22% [8,9,10]. 

In parotid carcinoma, neck dissection (ND) is recommended in patients with nodal metastases at presentation while elective management of the neck is considered in those with locally advanced diseases and those with high-grade pathologies [12,13,14,15,16,17,18,19]. While elective neck dissection (END) is usually not recommended in patients with early-stage and low-grade disease, there is significant variability in the rates of ND and the incidence of occult nodal metastases reported in the literature [12,13,14,15,16,17,18,19].

The objective of this study was to determine the frequency of occult nodal metastases in patients with acinic cell carcinoma of the parotid gland and secondarily to evaluate the value of the elective neck dissection END in regards to local control, disease-specific survival (DSS), and disease-free survival (DFS).

## 2. Materials and Methods 

A retrospective chart review of 66 patients with acinic cell carcinoma of the parotid gland was conducted at the Department of Otorhinolaryngology, Head and Neck Surgery of the Medical University of Vienna, the Institute of Head and Neck Diseases, Evangelical Hospital Vienna between 1998 and 2017 and the Princess Margaret Cancer Center, University Health Network, Toronto between 1989 and 2005. Patients with carcinoma of the submandibular or minor salivary glands, lymphoma or metastasis to the salivary glands were excluded. Patients were also excluded if they were previously treated elsewhere or insufficient data were retrievable. Demographic, clinical, and pathological data were retrieved from hospital records. Research Ethics Boards EK: 892/2017 and REB 17-5321 approved this present study.

### 2.1. Pathology

Diagnosis of all tumors followed the classification system for salivary gland malignancies of the World Health Organization (WHO) and was performed by experienced head and neck pathologists [1]. The following pathological data were obtained from pathologic reports; tumor grading, primary tumor (T-classification) and lymph node metastasis (N-classification), perineural and lymphovascular invasion (PNI and LVI), the status of resection margin, extraparotid extension, and metastasis to the intra- or periparotid lymph nodes. The grade was recorded as well-differentiated, moderately differentiated, and poorly differentiated and regrouped for analysis as low (G1), intermediate (G2), and high (G3) grade [2,3]. Periparotid lymph nodes were defined as nodes located in the immediate vicinity to the parotid gland. The resection margin was classified as being either tumor-free (R0) or positive (R1) in case of tumor tissue within 1 mm of the resection margin or a positive margin. Tumor staging was performed according to the eighth edition of the Union Internationale Contre le Cancer (UICC) classification system. 

### 2.2. Statistical Methods

Statistical analysis of data was performed using SPSS software (version 21; IBM SPSS Inc., Armonk, NY, USA). Clinicopathological data and incidence of nodal metastases were summarized using descriptive statistics. Data are indicated as mean ± standard deviation (SD) within result section. The Chi-square test was used to compare nominal variables and correlations were calculated with the contingency coefficient. Outcomes of interest included overall survival (OS; time period from date of surgery until last follow-up or death of any cause of the patient), disease-specific survival (DSS; defined as death from acinic cell tumor) and the disease-free survival (DFS; time period from date of surgery after clear surgical resection (R0) until the date of recurrence). Kaplan–Meier survival analysis and log-rank test were used to analyze OS, DSS, and DFS. Univariable analyses were performed to assess the prognostic impact of the following factors on DFS and DSS: T classification (T1–2 vs. T3–4), grading (G1 vs. G2 and G3), lymph node involvement (N0 vs. N+), PNI (PNI 1 vs. PNI 0), LVI (LVI 1 vs. LVI 0), resection margin status (R0 vs. R1), extraparotid extension (Yes vs. No), metastases to the periparotid lymph nodes (Yes vs. No), postoperative adjuvant radiotherapy (PORT Yes vs. No), and age (≥50 years vs. <50 years). Patients were dichotomized into a younger and older cohort using a median age of 50 years. Hazard Ratios (HR) with corresponding 95% confidence intervals (CI) are indicated. Given the limited number of patients and outcome events of interest in this study, multivariable analyses were not performed. All tests were performed two-sided, and *p*-values ≤ 0.05 were considered statistically significant. 

## 3. Results

### 3.1. Patients’ Clinical Data

There were 66 patients eligible for inclusion of which 34 (51.5%) were female and 32 (48.5%) male. Mean and median age was 49.4 and 50 years (range 16 years–91 years), respectively. Eight out of 66 patients (12.1%) presented with clinical and radiographic suspicious nodes (cN+), while there were no patients with distant metastases at presentation. The tumor characteristics and clinicopathological variables, including stage, grade, PNI, and LVI, are summarized in Table 1.

Parotidectomy with preservation of the facial nerve was performed in 90.9% (*n* = 60) of patients with six patients requiring facial nerve sacrifice, one of which also required temporal bone resection. Clear resections margins (R0) were achieved in 47 (71.2%) patients. Intermediate- or high-grade tumors were associated with significantly lower complete resection rates compared to low-grade tumors (78.4% vs. 37.5%; *p* = 0.015). Conversely, pT-classification (*p* = 0.247), PNI (*p* = 0.075), LVI (*p* = 0.107), extraparotid extension (*p* = 0.947), and involvement of periparotid lymph nodes (*p* = 0.573) had no significant impact on completeness of tumor resection. 

**Figure 1:**
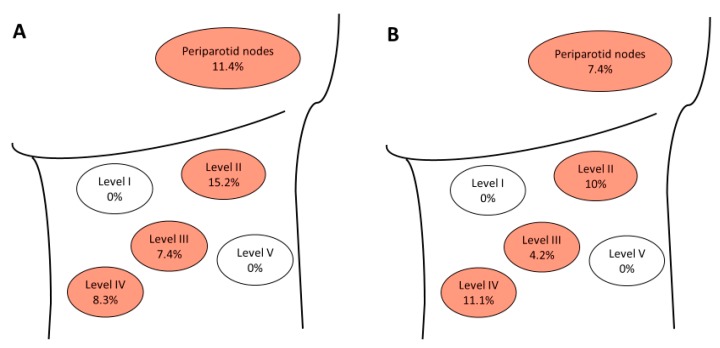
Distribution of locoregional lymph node metastases. Positive neck nodes of all patients (*n* = 35) undergoing neck dissection (**A**) and distribution of occult metastases (*n* = 27) in the neck after elective neck dissection (**B**). Levels I–V: percentage of involved nodes at that level.

Neck Dissection was performed in 35 (53%) patients, with END being performed in 27 patients (23 pT1/pT2 and four pT3/pT4 tumors), and a therapeutic ND in eight patients. The mean number of excised lymph nodes in the END group was 25 (±22) and 40.3 (±31.3) in the therapeutic neck dissection group; however, the difference was not statistically significant (*p* = 0.149). Overall, 12 (34.3%) out of 35 patients that underwent neck dissection had pathologic regional nodal metastases (N+). Occult regional lymph node metastases were found in four (14.8%) patients after END. The distribution of the nodes for the entire cohort, as well as those with the occult disease, is presented in Figure 1.

Pathological T classification (*p* = 0.087) tumor grade (*p* = 0.871), PNI (*p* = 0.396), LVI (*p* = 0.318), and extraparotid extension (*p* = 0.184) were not significantly associated with the presence of nodal metastases (Table 2).

Of note, two patients that underwent parotidectomy without neck dissection had a positive periparotid lymph node identified. Of the eight patients who underwent therapeutic ND, neck node metastases were detected in six patients (75%). Postoperative radiotherapy (PORT) was performed in 38 (57.6%) patients with a mean radiation dose of 58.6 Gy (median: 60 Gy; range 50 to 66 Gy). Adjuvant radiotherapy was used in 13 of 16 (81.3%) patients with positive margins, eight of nine (88.9%) patients with high-grade carcinoma, in all patients with perineural invasion, in nine of 12 (75%) patients with nodal metastases, in seven of 11 (63.6%) with T3 and T4 tumors, or where uncertainty existed about completeness of resection, usually arising from very close juxtaposition of the tumor to the facial nerve. 

### 3.2. Recurrence and Survival 

The mean (median) follow up time for all patients was 55.5 (46.7) months. During the study period, seven (10.6%) patients experienced recurrent disease (four local recurrences, one distant recurrence, one regional/distant recurrence, and one local/distant recurrence). The five-year local, regional, and distant control rates were 92.2%, 97.6%, and 94.2%, respectively. PNI (*p* = 0.010) and high-grade tumors were significantly associated with any recurrence (*p* = 0.002). Of note, three (42.9%) patients with a low-grade tumor, despite the use of an adjuvant PORT, had recurrent carcinoma. There was a trend, although not statistically significant, towards recurrent disease in carcinomas with LVI (*p* = 0.070) and in patients with positive resection margin (*p* = 0.094) (Table 3).

The five-year OS, DSS, and DFS rates were 92.9%, 96.2%, and 88.5%, respectively. At the time of the last follow-up, 57 (86.4%) patients were alive without disease and 3 (4.5%) with the disease. Six patients died (9.1%) of which three died of disease, and three died of other causes. Kaplan–Meier survival analyses were performed to determine the influence of the noted variables on OS, DSS, and DFS (Table 4). 

**Figure 2:**
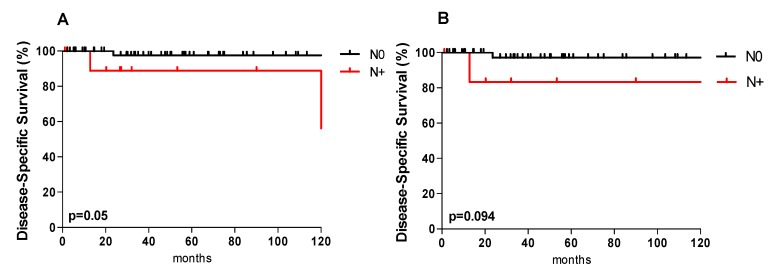
Disease-specific survival and nodal involvement. Kaplan–Meier disease-specific survival curves according to nodal involvement (N+ vs. N0) in the entire cohort (**A**) and for patients with T1 and T2 tumors (**B**).

Regarding OS, only LVI had a significant impact on OS (*p* = 0.004). The presence of lymph node metastases was significantly associated with a lower DSS rate compared to N0 patients (*p* = 0.05; Figure 2A). 

Subgroup analysis of only early stage (pT1/T2) patients with positive compared to negative neck nodes showed a worse but not significantly different DSS (*p* = 0.094; Figure 2B). Additional factors of worse DSS and DFS included intermediate to high-grade tumors, PNI and LVI (*p* = 0.007, *p* = 0.001 and *p* = 0.002, respectively) and (*p* = 0.002, *p* = 0.001, and *p* = 0.002, respectively). END had no significant benefit regarding OS, DSS and DFS (*p* = 0.795, *p* = 0.307, and *p* = 0.312).

On univariable cox regression analysis, only G1 tumor grade (HR 0.07; 95%CI: 0.01–0.85; *p* = 0.036) was a significant prognostic factor for DSS. Significant predictors of DFS included high-grade tumors (HR 8.62; 95%CI: 1.69–44.01; *p* = 0.010), PNI (HR 19.6; 95%CI: 0.01–0.37; *p* = 0.003), and LVI (HR 10.2; 95%CI: 0.02–0.59; *p* = 0.011). The influence of grading on the different clinicopathological factors is summarized in Table 5.

## 4. Discussion

The heterogeneous nature of malignancies of the parotid gland, along with their distinct biological behavior and clinicopathological characteristics, represents a significant challenge in management [1,2,3,4]. To date, tumor resection followed by END is strongly recommended for carcinomas of the parotid gland in cases of advanced-stage disease, high-grade tumor, present facial palsy, and local tissue invasion [14,15,16,17,18,19,20,21]. However, therapeutic algorithms in regards to low-grade malignancies of the parotid gland, such as acinic cell carcinomas, are variable. In particular, the reported frequency of positive neck nodes in acinic cell carcinoma of the parotid gland ranges between 0 and 43% [22,23,24,25]. Thus, we sought to study nodal metastases in a disease that is generally felt to be at low risk of nodal disease. In our study, lymph node metastases were detected in 34.3% of all specimens that underwent neck dissection, accounting for an overall incidence of 18.2% for the entire sample. Specifically, examining the rate of occult lymph node metastases, a National Cancer Data Base review reported occult metastases in cervical nodes in 22% of acinic cell carcinomas [8]. In our study, the rate of occult lymph node metastases tumors was 14.8%, which were predominately G1 acinic cell carcinomas.

Recurrence rates in our series were lower than what has been reported in the literature, with reported rates of local recurrence between 12.8 to 33% compared to our local recurrence rate of 7.6%. Similarly, the reported rates of regional recurrence in the literature range from 4.9 to 7.5%, which is higher compared to our regional recurrence rate of 1.5%. A possible explanation could be given by the high rate of elective neck treatment for our cohort (46.6%). Moreover, the indicated PORT rate of 57.6% is higher in comparison to the literature with reported rates of PORT for acinic cell carcinoma being between 37.1% and 52.9% [9,15,16] and the indications complied with the recent NCCN recommendations [19]. The distant metastases rate in our series of 4.5% is comparable to other cases series of acinic cell carcinoma [10,24].

Nodal status was a significant predictor of DSS with five-year DSS being 97.6% in node-negative patients compared to 88.9% in node-positive patients. This observation agrees with data of the National Cancer Data Base reporting on five-year OS of 90% in N0 patients with acinic cell carcinomas, which decreased to only 54% in N+ patients [8]. Similar to other parotid malignancies presence of nodal metastases in acinic cell carcinoma is associated with a worse prognosis. While END could help identifying patients with occult nodal metastases that subsequently carry a higher likelihood of worse outcome and may benefit from more aggressive treatment regimes, the overall incidence is low, and the majority of patients would not benefit from neck dissection. Particular attention should be paid to the peri-parotid nodes and the level II nodes, and if nodal metastases are identified during the parotidectomy an elective neck dissection should be performed. Thus, it is important to consent patients for the possibility of neck dissection preoperatively. The tumor grade, LVI, and PNI were significant predictors of DFS and should be factored into decision making regarding adjuvant therapy.

The major drawback of this study is its retrospective nature and an inherent bias due to the selection of information and its long observation period. Early in the time series, the distance of margins was not reported exactly in all patients. This represents an important information to determine if acinic cell carcinomas with close margins had the same outcome and oncological behavior as carcinomas with negative margins, as reported for adenoid cystic carcinoma [26]. Due to the rarity of this malignancy and a subsequently small number of patients with regional occult metastases this study lacks the power to prove *(i)* the definite benefit of END and *(ii)* to reveal occult metastases as an independent worse prognostic factor. However, based on the observed incidence of regional nodal metastases and in particular of occult regional lymph node metastases, an END should be considered for each patient with an acinic cell carcinoma of the parotid gland. However, for us, it seems evident that a higher rate of performed END will ultimately result in a greater percentage of detected occult lymph node metastases—a tendency that could be found in other studies [9,12,15,16]. As a result, further efforts need to be made, with the help of greater multicentric prospective studies, to validate further the survival benefit for patients with acinic cell carcinomas of the parotid gland undergoing elective neck dissection.

## Figures and Tables

**Table 1:** Demographic and clinicopathological data of 66 patients with acinic cell carcinoma of the parotid gland.

Clinical Variables		Nr.
**Age, years (mean ± SD)**		49.4 ± 17.1
**Sex**		
	Male	32 (48.5%)
	Female	34 (51.5%)
**pT classification**		
	T1	30 (45.5%)
	T2	25 (37.9%)
	T3	9 (13.6%)
	T4	2 (3%)
**N classification**		
	N0	54 (81.8%)
	N+	12 (18.2%)
**Staging**		
	I	27(40.9%)
	II	19 (28.8%)
	III	12 (18.2%)
	IV	4 (6.1%)
	n.a.	4 (6%)
**Tumor Grading**		
	Low-grade (G1)	53 (80.3%)
	Intermediate-grade (G2)	3 (4.5%)
	High-grade (G3)	6 (9.1%)
	n.a.	4 (6.1%)
**Perineural Invasion**		
	negative (PNI 0)	53 (80.3%)
	positive (PNI 1)	4 (6.1%)
	n.a.	9 (13.6%)
**Lymphovascular Invasion**		
	negative (LVI 0)	51 (77.3%)
	positive (LVI 1)	7 (10.6%)
	n.a.	8 (12.1%)
**Extraparotid Extension**		
	negative	54 (81.8%)
	positive	4 (6.1%)
	n.a.	8 (12.1%)
**Periparotid lymph node involvement**		
	negative	45 (68.2%)
	positive	5 (7.6%)
	n.a.	16 (24.2%)

Nr., number of patients; n.a., not available.

**Table 2:** Clinicopathological variables affecting lymph nodes metastasis.

Variables		Total	N Classification			
			N+	Occult N	N0	*p* ^a^
**pT classification**						
	pT1–pT2	55	8 (14.5%)	4 (7.3%)	47 (85.5%)	
	pT3–pT4	11	4 (36.4%)	2 (18.2%)	7 (63.6%)	0.087
**Tumor Grading**						
	G1	53	9 (17%)	4 (7.3%)	44 (83%)	
	G2-G3	9	2 (22.2%)	1 (11.1%)	7 (77.8%)	
	n.a.	4	1 (25%)	1 (25%)	3 (75%)	0.871
**PNI**						
	negative	53	8 (15.1%)	3 (5.7%)	45 (84.9%)	
	positive	4	1 (25%)	1 (25%)	3 (75%)	
	n.a.	9	3 (33.3%)	2 (22.2%)	6 (66.7%)	0.396
**LVI**						
	negative	51	8 (15.7%)	3 (5.9%)	43 (84.3%)	
	positive	7	1 (14.3%)	1 (14.3%)	6 (85.7%)	
	n.a.	8	3 (37.5%)	2 (25%)	5 (62.5%)	0.318
**Extraparotid extension**						
	negative	54	8 (14.8%)	3 (5.6%)	47(85.2%)	
	positive	4	2 (50%)	1 (25%)	2 (50%)	
	n.a.	8	2 (25%)	2 (25%)	6 (75%)	0.184

*p*, *p*-value; ^a^ Chi-square test was performed between N+ and N0; n.a., not available; PNI, perineural invasion; LVI, lymphovascular invasion.

**Table 3:** Clinicopathological variables and recurrence.

Variables		Recurrence		*p* ^a^
		YES	NO	
		Nr. (%)	Nr. (%)	
**pT classification**				
	pT1–pT2	5 (71.4%)	50 (84.7%)	
	pT3–pT4	2 (28.6%)	9 (15.3%)	0.371
**Occult metastasis**				
	yes	0	6 (10.2%)	
	no	7 (100%)	53 (89.8%)	0.376
**N classification**				
	N0	1 (14.3%)	24 (40.7%)	
	pN+	2 (28.6%)	10 (16.9%)	
	no ND	4 (57.1%)	25 (42.4%)	0.382
**Neck dissection**				
	yes	3 (42.9%)	32 (54.2%)	
	no	4 (57.1%)	27 (45.8%)	0.568
**Grading**				
	G1	3 (42.9%)	50 (84.7%)	
	G2–G3	4 (57.1%)	5 (8.5%)	
	n.a.	0	4 (6.8%)	0.002
**Margin status**				
	negative (R0)	3 (42.9%)	44 (74.6%)	
	positive (R1)	4 (57.1%)	12 (20.3%)	
	n.a.	0	3 (5.1%)	0.094
**PNI**				
	negative	3 (42.9%)	50 (84.7%)	
	positive	2 (28.6%)	2 (3.4%)	
	n.a.	2 (28.6%)	7 (11.9%)	0.010
**LVI**				
	negative	3 (42.9%)	48 (81.4%)	
	positive	2 (28.6%)	5 (8.5%)	
	n.a.	2 (28.6%)	6 (10.1%)	0.070
**Extraparotid extension**				
	negative	6 (85.7%)	48 (81.4%)	
	positive	1 (14.3%)	3 (5.1%)	
	n.a.	0	8 (13.6%)	0.399
**Periparotid lymph node involvement**				
	negative	5 (71.4%)	40 (67.8%)	
	positive	1 (14.3%)	4 (6.8%)	
	n.a.	1 (14.3%)	15 (25.4%)	0.671
**PORT**				
	yes	6 (85.7%)	32 (54.2%)	
	no	1 (14.3%)	27 (45.8%)	0.111

*p*, *p*-value; ^a^ Chi-square test; n.a., not available; PNI, perineural invasion; LVI, lymphovascular invasion; PORT, postoperative radiotherapy.

**Table 4:** Kaplan–Meier survival analyses.

Variables		Overall Survival			Disease-Specific Survival			Disease-Free Survival		
		1 y	5 y	*p* ^a^	1 y	5 y	*p* ^a^	1 y	5 y	*p* ^a^
**pT classification**										
	pT1–pT2	98.2	91.3		100	95.3		91.9	91.7	
	pT3–pT4	100	100	0.126	100	100	0.505	100	75.0	0.329
**N classification**										
	N0	100	95.4		100	97.6		93.8	91.3	
	N+	91.7	81.5	0.109	100	88.9	0.050	90	67.5	0.182
**Margin status**										
	negative (R0)	97.9	93.0		100	97.6		95.5	92.6	
	positive (R1)	100	90.9	0.244	100	90.9	0.406	85.1	70.9	0.210
**Grading**										
	G1	98.1	95.9		100	100		95.7	93	
	G2–G3	100	68.6	0.148	100	68.6	0.007	72.9	48.6	0.002
**PNI**										
	no	98.1	95.9		100	100		95.9	93.3	
	yes	100	66.7	0.063	100	66.7	0.001	66.7	0	0.001
**LVI**										
	no	100	97.7		100	100		97.9	92.3	
	yes	85.7	64.3	0.004	100	75.0	0.002	75.0	50.0	0.002
**PORT**										
	no	96.4	91.6		100	100		100	100	
	yes	100	93.7	0.832	100	93.7	0.180	88.4	81.0	0.038
**END**										
	no	100	91.5		100	95.5		92.3	87.2	
	yes	96.6	96.6	0.795	100	100	0.307	96.2	96.2	0.312

*p*, *p*-value; y, years; ^a^ Log-rank test; PNI, perineural invasion; LVI, lymphovascular invasion; PORT, postoperative radiotherapy; END, elective neck dissection.

**Table 5:** Comparison of low grade and intermediate/ high grade acinic cell carcinoma of the parotid gland.

Variables		Grading		*p* ^a,b^
		G1	G2–G3	
		Nr. (%)	Nr. (%)	
**pT classification**				
	pT1–pT2	45 (84.9%)	6 (66.7%)	
	pT3–pT4	8 (15.1%)	3 (33.3%)	0.185 ^a^
**N classification**				
	N0	23 (43.4%)	1 (11.1%)	
	pN+	9 (17%)	2 (22.2%)	
	no ND	21 (39.6%)	6 (66.7%)	0.175 ^a^
**Neck dissection**				
	yes	31 (58.5%)	3 (33.3%)	
	no	22 (41.5%)	6 (66.7%)	0.161 ^a^
**Margin status**				
	negative (R0)	40 (75.5%)	3 (33.3%)	
	positive (R1)	11 (20.8%)	5 (55.6%)	
	n.a.	2 (3.7%)	1 (11.1%)	0.040 ^a^
**PORT**				
	yes	28 (52.8%)	8 (88.9%)	
	no	25 (47.2%)	1 (11.1%)	0.043 ^a^
**Overall Survival**				
	1 year	98.1	100	
	5 year	95.9	68.6	0.148 ^b^
**Disease-specific survival**				
	1 year	100	100	
	5 year	100	68.6	0.007 ^b^
**Disease-free survival**				
	1 year	95.7	72.9	
	5 year	93	48.6	0.002 ^b^
**PNI**				
	negative	49 (92.5%)	3 (33.3%)	
	positive	0 (0%)	4 (44.5%)	
	n.a.	4 (7.5%)	2 (22.2%)	0.001 ^a^
**LVI**				
	negative	44 (83.1%)	5 (55.6%)	
	positive	5 (9.4%)	2 (22.2%)	
	n.a.	4 (7.5%)	2 (22.2%)	0.169 ^a^
**Extraparotid extension**				
	negative	45 (84.9%)	8 (88.9%)	
	positive	3 (5.7%)	1 (11.1%)	
	n.a.	5 (9.4%)	0 (0%)	0.544 ^a^
**Periparotid lymph node involvement**				
	negative	35 (66.1%)	9 (100%)	
	positive	5 (9.4%)	0 (0%)	
	n.a.	13 (24.5%)	0 (0%)	0.116 ^a^

Data of four patients are excluded due to missing grading information; *p*, *p*-value; ^a^ Chi-square test; ^b^ Log-rank test; n.a., not available; PNI, perineural invasion; LVI, lymphovascular invasion; PORT, postoperative radiotherapy.

## References

[1] Seifert G., Sobin L.H. (1991). Histological typing of salivary gland tumours. World Health Organization International Histological Classification of Tumours.

[2] Hoffman H.T., Karnell L.H., Robinson R.A., Pinkston J.A., Menck H.R. (1999). National Cancer Data Base report on cancer of the head and neck: Acinic cell carcinoma. Head Neck.

[3] Vander Poorten V., Bradley P.J., Takes R.P., Rinaldo A., Woolgar J.A., Ferlito A. (2012). Diagnosis and management of parotid carcinoma with a special focus on recent advances in molecular biology. Head Neck.

[4] Spiro R.H. (1986). Salivary neoplasms: Overview of a 35-year experience with 2807 patients. Head Neck.

[5] Erovic B.M., Schopper C., Pammer J., Vormittag L., Maleki A., Brunner M., Heiduschka G., Grasl M.C., Thurnher D. (2010). Multimodal treatment of patients with minor salivary gland cancer in the case of recurrent disease. Head Neck.

[6] Haymerle G., Schneider S., Harris L., Häupl T., Schopper C., Pammer J., Grasl M.C., Erovic B.M. (2016). Minor salivary gland carcinoma: A review of 35 cases. Eur. Arch. Otorhinolaryngol..

[7] Schneider S., Kloimstein P., Pammer J., Brannath W., Grasl M.C., Erovic B.M. (2014). New diagnostic markers in salivary gland tumors. Eur. Arch. Otorhinolaryngol..

[8] Xiao C.C., Zhan K.Y., White-Gilbertson S.J., Day T.A. (2016). Predictors of nodal metastasis in parotid malignancies: A National Cancer Data Base Study of 22,653 Patients. Otolaryngol. Head Neck Surg..

[9] Scherl C., Kato M.G., Erkul E., Graboyes E.M., Nguyen S.A., Chi A.C., Morgan P.F., Day T.A. (2018). Outcomes and prognostic factors for parotid acinic cell Carcinoma: A National Cancer Database study of 2362 cases. Oral. Oncol..

[10] Ellis G.L., Corio R.L. (1983). Acinic cell adenocarcinoma. A clinicopathologic analysis of 294 cases. Cancer.

[11] Erovic B.M., Shah M.D., Bruch G., Johnston M., Kim J., O’Sullivan B., Perez-Ordonez B., Weinreb I., Atenafu E.G., de Almeida J. (2015). Outcome analysis of 215 patients with parotid gland tumors: A retrospective cohort analysis. J. Otolaryngol. Head Neck Surg..

[12] Armstrong J.G., Harrison L.B., Thaler H.T., Friedlander-Klar H., Fass D.E., Zelefsky M.J., Shah J.P., Strong E.W., Spiro R.H. (1992). The indications for elective treatment of the neck in cancer of the major salivary glands. Cancer.

[13] Kawata R., Koutetsu L., Yoshimura K., Nishikawa S., Takenaka H. (2010). Indication for elective neck dissection for N0 carcinoma of the parotid gland: A single institution’s 20-year experience. Acta Otolaryngol..

[14] Eneroth C.M., Hamberger C.A. (1974). Principles of treatment of different types of parotid tumors. Laryngoscope.

[15] Ali S., Palmer F.L., DiLorenzo M., Shah J.P., Patel S.G., Ganly I. (2014). Treatment of the neck in carcinoma of the parotid gland. Ann. Surg. Oncol..

[16] Stenner M., Molls C., Luers J.C., Beutner D., Klussmann J.P., Huettenbrink K.B. (2012). Occurrence of lymph node metastasis in early-stage parotid gland cancer. Eur. Arch. Otorhinolaryngol..

[17] Medina J.E. (1998). Neck dissection in the treatment of cancer of major salivary glands. Otolaryngol. Clin. N. Am..

[18] Zbaren P., Schupbach J., Nuyens M., Stauffer E. (2005). Elective neck dissection versus observation in primary parotid carcinoma. Otolaryngol. Head Neck Surg..

[19] Colevas A.D., Yom S.S., Pfister D.G., Spencer S., Adelstein D., Adkins D., Brizel D.M., Burtness B., Busse P.M., Caudell J.J. (2018). NCCN Guidelines Insights: Head and Neck Cancers, Version 1.2018. J. Natl. Compr. Cancer Netw..

[20] Mc Guirt W.F. (1989). Management of occult metastatic disease from salivary gland neoplasms. Arch. Otolaryngol. Head Neck Surg..

[21] Kelley D., Spiro R. (1996). Management of the neck in parotid carcinoma. Am. J. Surg..

[22] Gomez D.R., Katabi N., Zhung J., Wolden S.L., Zelefsky M.J., Kraus D.H., Shah J.P., Wong R.J., Ghossein R.A., Lee N.Y. (2009). Clinical and pathologic prognostic features in acinic cell carcinoma of the parotid gland. Cancer.

[23] Eneroth C.M., Jakobsson P.A., Blanck C. (1966). Acinic cell carcinoma of the parotid gland. Cancer.

[24] Spiro R.H., Huvos A.G., Strong E.W. (1978). Acinic cell carcinoma of salivary origin. A clinicopathologic study of 67 cases. Cancer.

[25] Lin W.N., Huang H.C., Wu C.C., Liao C.T., Chen I.H., Kan C.J., Huang S.F. (2010). Analysis of acinic cell carcinoma of the parotid gland—15 years experience. Acta Otolaryngol..

[26] Amit M., Na’ara S., Trejo-Leider L., Ramer N., Burstein D., Yue M., Miles B., Yang X., Lei D., Bjoerndal K. (2017). Defining the surgical margins of adenoid cystic carcinoma and their impact on outcome: An international collaborative study. Head Neck.

